# EFFECTIVENESS OF A CLASSIFICATION-BASED APPROACH TO LOW BACK PAIN IN PRIMARY CARE: A BENCHMARKING CONTROLLED TRIAL

**DOI:** 10.2340/jrm.v56.28321

**Published:** 2024-04-20

**Authors:** Anna Sofia SIMULA, Antti MALMIVAARA, Neill BOOTH, Jaro KARPPINEN

**Affiliations:** 1 Medical Research Center Oulu, Oulu University Hospital and University of Oulu, Oulu, Finland; 2 Department of General Medicine, Wellbeing services county of South Savo (ELOISA), Mikkeli, Finland; 3 Finnish Institute for Health and Welfare, Helsinki, Finland; 4 Orton Orthopaedic Hospital, Helsinki, Finland; 5 Faculty of Social Sciences (Health Sciences), Tampere University, Tampere, Finland; 6 Rehabilitation Services of Wellbeing Services County of South Karelia, Lappeenranta, Finland

**Keywords:** low back pain, classification, patient education, primary care, benchmarking

## Abstract

**Objective:**

The aim of this study was to assess the effectiveness of classification-based approach for low back pain care in Finnish primary care.

**Design:**

A benchmarking controlled trial design was used.

**Subjects/patients:**

Three primary healthcare areas and 654 low back pain patients with or without sciatica.

**Methods:**

Classification-based care (using the STarT Back Tool) was implemented using organizational-, healthcare professional-, and patient-level interventions. The primary outcome was change in Patient-Reported Outcomes Measurement Information System, Physical Function (PROMIS PF-20) from baseline to 12 months.

**Results:**

No difference was found between the intervention and control in change in PROMIS PF-20 over the 12-month follow-up (mean difference 0.33 confidence interval –2.27 to 2.9, *p* = 0.473). Low back pain-related healthcare use, imaging, and sick leave days were significantly lower in the intervention group. Reduction in intensity of low back pain appeared to be already achieved at the 3-month follow-up (mean difference –1.3, confidence interval –2.1 to –0.5) in the intervention group, while in the control group the same level of reduction was observed at 12 months (mean difference 0.7, confidence interval –0.2 to 1.5, treatment*time *p* = 0.003).

**Conclusion:**

Although classification-based care did not appear to influence physical functioning, more rapid reductions in pain intensity and reductions in healthcare use and sick leave days were observed in the intervention group.

LAY ABSTRACT

Low back pain is a leading cause of disability globally and a major economic burden for healthcare systems and societies. The aim of this study was to find out whether a classification-based approach to low back pain in primary care is effective in improving physical functioning or in reducing healthcare use compared with the usual care. Healthcare professionals in three primary healthcare area were educated to provide classification-based care and a patient education booklet was offered to support appropriate care. Although no difference was observed in brief generic health measure (PROMIS-20, the primary outcome) over the 12-month follow-up, more rapid reductions in pain intensity and in healthcare use was observed in the intervention group. A primary-care focused intervention seems to decrease costs related to low back pain.

Low back pain (LBP) is a common symptom (1), and tends to reoccur causing disability and work absence (1, 2). Unfortunately, guideline recommendations to remain active, stay at work, to consider psychosocial aspects and provide education and advice are incompletely implemented in practice (3–5). Overtreatment and low-value care for LBP seem commonplace (6, 7). For example, inappropriate imaging for LBP is associated with additional invasive procedures, work disability, and increased healthcare use and costs (8, 9). A patient education booklet supports evidence-based care for LBP, improving recovery, decreasing pain-related fear, encouraging physical activity, and reducing inappropriate imaging and work disability (10, 11). Classification-based care of patients with LBP according to the STarT Back Tool (SBT) in primary care has been shown to be effective, halving time off work without increasing healthcare costs (2, 12, 13).

The aim of this study was to assess the short- (over the first 3 months) and long-term (over the first year, primary outcome) effectiveness of implementation of a classification-based approach including education of primary care professionals, in terms of physical functioning, quality of life, and work disability among patients with LBP (14). Furthermore, we assessed LBP-related healthcare use in comparison with usual care.

## METHODS

### Study setting

**Fig. 1:**
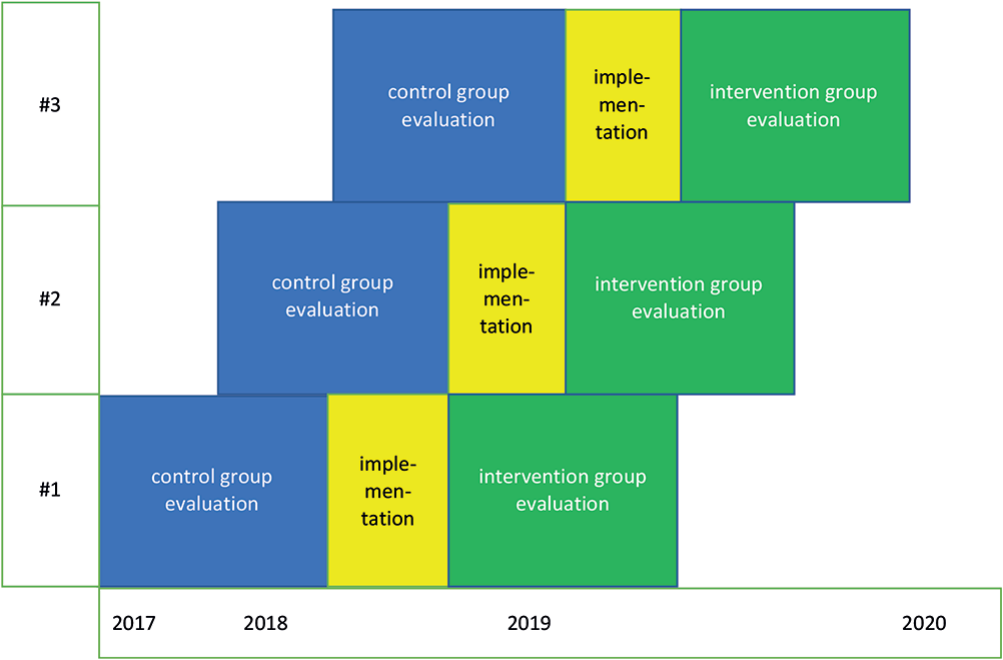
Organisational-level flow of the benchmarking controlled trial. #1 ESSOTE (Etelä-Savon sosiaali- ja terveystoimi, the South Savo social and health care authority, population 80,000); #2 EKSOTE (Etelä-Karjalan sosiaali- ja terveyspiiri, South Karelia social and health care district, population 129,000); #3 the City of Rovaniemi (population 60,000).

We used a benchmarking controlled trial to assess the effectiveness of a classification-based approach to LBP in primary care (Fig. 1) (15, 16). Ethics approval was granted by the Ethics Committee of the University Hospital of Oulu and the study protocol followed the Declaration of Helsinki. The trial was registered at ISRCTN (ISRCTN13273552, registered 13.5.2019).

### Participants

Three primary health care areas went through 3 phases: (i) evaluation before implementation (control group), (ii) implementation of a classification-based approach, and (iii) evaluation after implementation (intervention group) (Fig. 1). Patients in the control and intervention groups were all different individuals. The leaders of all the participating healthcare areas gave permission for the study.

All healthcare professionals (HCPs) including physicians, physiotherapists, and nurses) who were involved in treatment of patients with LBP in primary care were invited to participate in the study and to recruit eligible patients. Educational training was offered to all invited HCPs even if they did not recruit patients.

Eligibility criteria included patients aged 18–65 who contacted primary healthcare due to LBP with or without radicular pain. Exclusion criteria were: (i) age under 18 or over 65; (ii) first patient-reported healthcare contact due to LBP and episode lasting less than 2 weeks; (iii) suspicion of a serious cause of LBP or LBP requiring urgent care. Signed consent was required and patients were free to discontinue their participation in the study at any time. Fig. S1 presents the patient-level study flow.

### Intervention

Detailed information on the intervention has been described elsewhere (14). Organizational-level interventions consisted of (*i*) developing premeditated care pathways for LBP patients according to SBT risk group, (ii) targeting more resources towards high-risk patients, and (iii) adding more adherence-supporting phrases to the electronic patient record (EPR) system. The intervention supported direct access to physiotherapy (access to physiotherapy without referral from a physician) for all LBP patients and, in addition, fast access to physiotherapy for high-risk patients bypassing possible usual waiting times. The intervention supported nurses to take part in enhanced patient care. Nurses in all healthcare areas also took part in patient care relating to other common diseases using supportive phrases as a checklist for anamnesis and other measures before physician appointments. We entered LBP-related supportive phrases for the nurses in the EPR system, thus ensuring that LBP patient received the patient education booklet, filled in the SBT and received referral to physiotherapy according to their SBT risk group. Organizational-level aspects of the intervention were customized to each organization in collaboration with local healthcare leaders.

The HCP-level intervention consisted of education and offering helpful tools (the educational booklet, use of the SBT, and phrases in the EPR system) to deliver classification-based interventions for the LBP patients. Physicians received 4-hour, physiotherapists 4-day, and nurses 2-hour education. In addition, some short booster education sessions were organized in the units during their weekly meetings. The full programme of the education sessions is available as supplementary material. The HCPs were taught to use the SBT as either part of a phrase in the EPR system or as a printed version, and to use it systematically with all LBP patients and to refer LBP patients to physiotherapy in accordance with their SBT risk-group classification. The HCPs were educated on the contents of the patient education booklet and encouraged to use the booklet with all LBP patients (10). One nurse was tasked in each organization with ensuring that printed booklets were available in every appointment room.

All LBP patients received the patient education booklet, were supposed to complete the SBT, and received biopsychosocial oriented care according to the individual SBT-based risk profile (12, 14, 17, 18). Medium-risk patients were referred to physiotherapy after 4 to 6 weeks. High-risk patients were supposed to receive psychologically informed physiotherapy after less than 1 week.

### Control

LBP patients received the usual care, which at that time meant limited direct access (because of minimal resources and broad exclusion criteria) to physiotherapy. Nurses did not systematically participate in LBP patient care before the physician appointment. The SBT was not part of the usual care in Finland at this time. HCPs’ education regarding psychosocial risk factors was sporadic. Referral to physiotherapy depended on the individual HCP. Typical delays in receiving physiotherapy after the first HCP contact were from 4 to 6 weeks.

### Patient-reported data

Patient-reported data at baseline, at 3 months, and at 1 year was collected by web-based questionnaires via email, or printed questionnaires if email addresses were missing. The research assistant emailed participants a link to the questionnaire after the first HCP contact and signed consent. If the questionnaire had still not been completed after a reminder email, the research assistant sent a text message with a hyperlink to the questionnaire at 2 and 3 weeks and confirmed by phone that the participant had received the questionnaire.

### Electronic patient record (EPR) data

The data were collected manually from the EPR by the first author and research assistant using a checklist for each patient (Table SI). The first 10 checklists were filled separately by 2 people and compared to ensure accuracy. The meaning of each word in the checklist was discussed, and a routine check of all documentation during the study follow-up, including the day of signed consent, was undertaken. After the first and second checklists were discussed, accuracy reached 100%. The data were collected from the date of signed consent to 3- and 12-month follow-ups. In Finland, EPR systems are connected to the nationwide database (Kanta), which is used in all primary and secondary healthcare organizations. Each HCP is obligated to record the health data from every healthcare contact on the system. All primary care HCPs (physicians, physiotherapists, nurses, etc.) can access the same EPR data system.

### Implementation fidelity

Implementation fidelity has been assessed using EPR data. The first-contacting professional is defined as the HCP that the patient contacted first, and to whom they gave their signed consent. The specific elements of classification-based LBP care recorded at first study contact were (i) the use of supportive phrases by a nurse in the EPR entry, (ii) the SBT risk group was assessed and documented, (iii) the LBP care plan was documented according SBT risk group, (iv) any psychosocial or lifestyle aspects were documented (mood, social circumstances, sleep quality, physical activity, and smoking), (v) any active treatment plan was documented (suggestions to stay active, training advice, or referral to physiotherapy).

### Outcomes

The primary outcome was change in Patient-Reported Outcomes Measurement Information System, Physical Functioning, short form 20a (PROMIS PF-20) from baseline to the 12-month follow-up (19, 20).

### Secondary outcomes

LBP-related healthcare use and number of sick leave days were evaluated over 3-month and 12-month follow-up using EPR data. LBP related healthcare use included physician visits in primary and secondary care, PT visits, nurse visits, and the proportion of patients who had undergone imaging examination (radiographs/MRI [magnetic resonance imaging]/CT [computed tomography]), and number of surgical interventions.

Secondary patient-reported outcomes were change in PROMIS PF-20 from baseline to 3-month follow-up; change in Oswestry Disability Index (ODI) (21), LBP and leg pain intensity during past week (0–100) (LBP VAS/ leg pain VAS) from baseline to 3- and 12-month follow-ups; change in EQ-5D-3L (EuroQol 5 dimensions) index; and self-rated health (0-100) from baseline to 12-month follow-up (22).

Pain-related baseline characteristics were evaluated at baseline using the Fear Avoidance Beliefs Questionnaire (FABQ) along with its subclassifications (23, 24), Pain Self-Efficacy Beliefs Questionnaire (PSEQ) (25), Ronald Morris Disability Questionnaire (RMDQ), and short form of Örebro Musculoskeletal Pain Screening Questionnaire (ÖMPSQ-short) total scores and subgroups (26–28). The frequency of pain medication use for LBP was recorded by using the dichotomous response, “yes” or “no”, to the question: “Have you used pain medication on three days or more during last week?”

### Sample size

Our sample size calculation is based on the following hypothesis to be tested: superiority of a classification-based approach to LBP in primary care compared with best current care with the primary outcome measure for this trial being PROMIS PF-20 from baseline to 12-month follow-up. According to sample size calculation for PROMIS PF-20 change, a sample size of 340 with 40% dropout was required for 80% power. For sample size calculation, we used G*Power 3.1) https://www.psychologie.hhu.de/arbeitsgruppen/allgemeine-psychologie-und-arbeitspsychologie/gpower) (difference between two dependent means). The minimal important difference (MID) for PROMIS PF-20 change was 2 points, the standard deviation (SD) was 3.66 (29). The effect size was 0.5, and type I error rate 0.05. A sample size of 34 patients per healthcare region would enable the detection of a difference of 2 points in PROMIS with 80% power. With a 40% dropout rate and 6 groups (3 healthcare regions before and 3 after), the final sample size needed is 340 patients. We received a sample size of 654 patients with 48% dropout, which exceeds the estimated sample size required for the primary outcome measure.

### Statistical methods

Between-group differences in baseline characteristics were analysed using independent-samples *t*-tests, the Mann–Whitney U tests and the χ^2^ test or Fisher’s exact tests. The differences between the intervention and control groups in the repeated (baseline, 3-month, 12-month) measures of continuous outcomes (PF-20, ODI, NRS for pain, EQ-5D index, EQ VAS, FABQ, PSEQ, RMDQ) were analysed using a linear model, which provided estimated least squares mean differences (MD) with a 95% confidence interval. Dichotomous outcomes (imaging, LBP frequency) were analysed using binary logistic regression, providing an estimated between-group difference expressed as an odds ratio (OR) with a 95% confidence interval (CI). Ordinal outcomes (SBT) were analysed using ordinal logistic regression. Poisson regression was used for count outcomes (sick leave days, healthcare appointments) providing an estimated between-group difference expressed as risk ratio (RR) with a 95% confidence interval. In all the regression analyses we used generalized estimating equations with an exchangeable working correlation matrix to consider clustered structure of the data. We used a full intention-to-treat method (which included adjustment for baseline measure of the respective outcomes) in all the repeated measures to assess the differences over time between the intervention and control.

We made additional adjusted analyses in three steps for the repeated measures, healthcare use, and number of sick leave days. In the first step, we adjusted for pain-unrelated confounders, including the number of comorbidities, smoking, age, and gender. The second step included adjustment for additional pain-related confounders: the presence of radicular pain, multisite pain (SBT question 2: “I have had pain in the shoulder or neck at some time in the last 2 weeks”), and chronic pain (LBP lasting 3 months or longer, modified variable from the Örebro-short question 1). In the third step, we adjusted the analysis using the SBT risk group from the baseline questionnaire.

## RESULTS

**Fig. 2:**
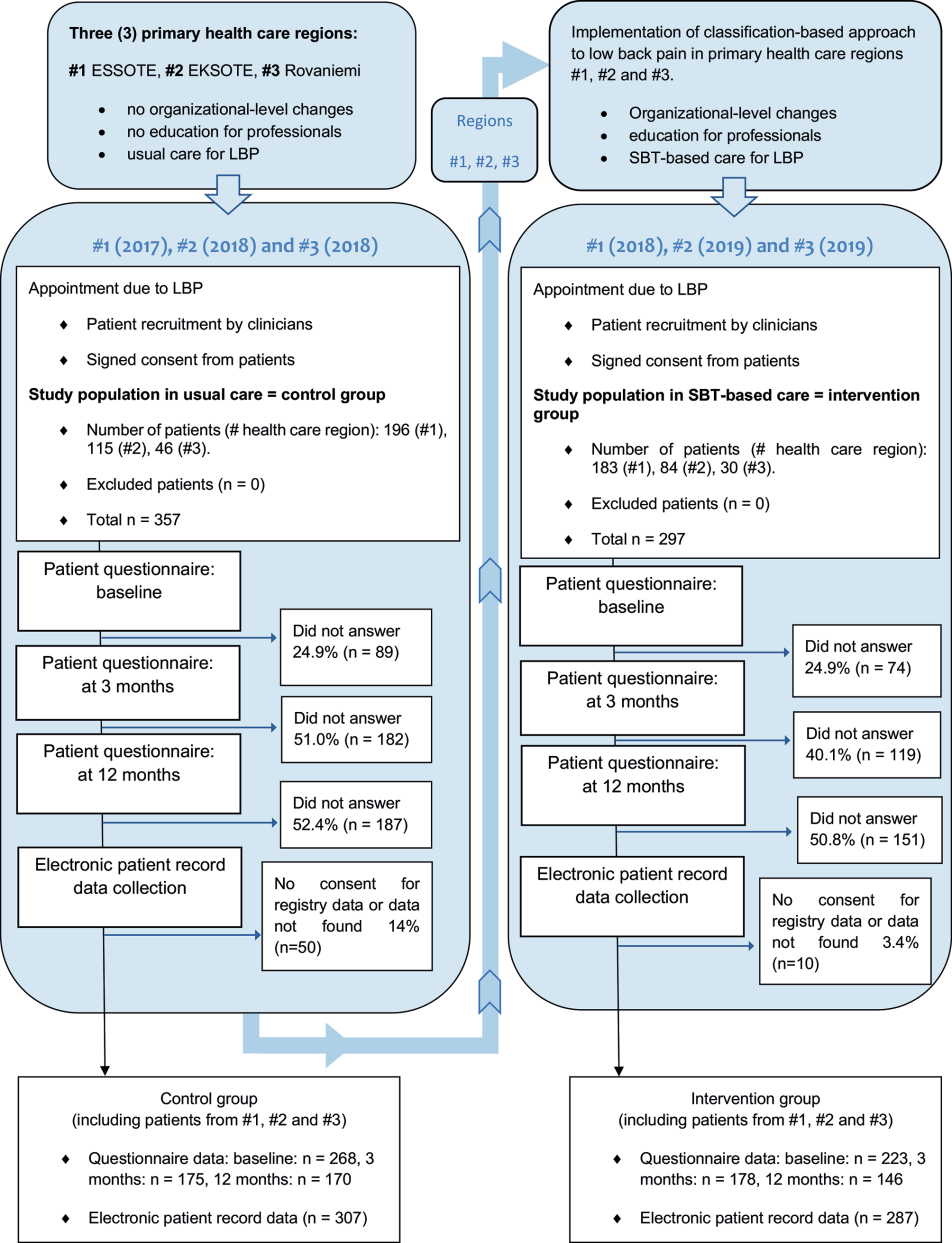
Trial flow diagram.

A total of 654 LBP patients consented to participate in the study (Fig. 2).

### Baseline characteristics

The patients in the intervention and control groups had similar baseline demographic data and patient-reported characteristics (Table I). Baseline characteristics after first visit to the HCP are patient-reported characteristics from the baseline questionnaire, which were potentially affected by the first visit to the HCP as they have been collected 1 to 3 weeks after the first contact. Implementation of the SBT-based approach to LBP has been carried out in the intervention group at this time point according to the instructions given to HCPs during training. Differences were seen after the first contact in pain-related fear, pain self-efficacy, ÖMPSQ-short (total scores and risk groups), pain medication use, pain intensity, pain-related disability, physical functioning, self-rated health, and LBP frequency.

**Table I:** Patient-reported baseline characteristics *c*.1–3 weeks after the first visit to healthcare professional. The visit was delivered as usual care in the control group and as classification-based care in the intervention group

Demographic data and long-term patient reported data	Intervention (*n*=297)	Control (*n*=357)	Mean difference (MD)^2^ (95% CI)*	*p*-value
Age^1^ (years)	43.6 (12.8)	43.0 (12.7)		0.546
Gender female^2^	62.6 (186)	61.6 (220)		0.793
Smoking^2^	26.9 (60)	29.1 (78)		0.589
Comorbidity^2^				
Diabetes	7.6 (17)	6.4 (17)		0.594
Rheumatoid arthritis	0.9 (2)	1.5 (4)		0.693
Spondylarthritis	0.9 (2)	0.4 (1)		0.594
Osteoarthritis	22.7 (53)	22.6 (60)		0.752
Depression	18.4 (41)	19.9 (53)		0.667
Fibromyalgia	4.5 (10)	2.6 (7)		0.265
Inflammatory bowel disease	9.4 (21)	3.4 (9)		0.006
Muscle disease	1.8 (4)	0.4 (1)		0.183
Number of comorbidities^3^	0 (1)	0 (1)		0.163
Multisite pain^2^	73.1(163)	68.3 (183)		0.245
Chronic LBP (> 3months)^2^	45.3(101)	45.1 (121)		0.975
Radicular pain % (*n*)^2^ (Patient-reported, SBT Question 1)	65.5 (146)	72.4 (194)		0.098
Body mass index^3^ (kg/m^2^)	26.8 (7.5)	27.2 (7.3)		0.517
Physically inactive^2^	4.5 (10)	3.5 (9)		0.519
(light exercise ≤ 1/month)				
DEPS score^3^	6 (8)	6 (9)		0.445
Actively working^2^	59.6 (133)	56.7 (152)		0.513
Work ability^3^ (0–10)	7 (3)	7 (3)		0.157
Baseline characteristics, which were potentially affected by the intervention in the intervention group	Mean^2^ (SE) Intervention	Mean^2^ (SE) Control	Mean difference (MD)^2^ (95% CI)*	*p*-value
Pain-related fear (FABQ)^4^	26.2(0.1)	29.5 (1.1)	–3.3 (0.01 to 0.29)	**0.002**
Pain-related fear (FABQ) – Work**^4^	13.9 (0.2)	16.1 (0.8)	–2.3 (0.02 to 0.49)	**0.004**
Pain-related fear (FABQ) – Physical activity***^4^	11.4 (0.2)	13.0 (0.2)	–1.7 (0.09 to 0.43)	**<0.001**
Pain Self-Efficacy beliefs Questionnaire (PSEQ)^4^	44.2 (0.3)	41.9 (0.3)	2.3 (3.3 to 29.9)	**<0.001**
Physical functioning (PROMIS PF-20 T-score)^4^	45.3 (0.1)	44.0 (0.2)	1.3 (2.0 to 6.2)	**<0.001**
Physical impairment, (RMDQ)^4^	7.3 (1.2)	6.7 (0.1)	0.66 (0.17 to 22.25)	0.597
Disability (ODI%)^4^	21.9 (0.3)	23.7 (0.4)	**–1.9 (–3.2 to –0.5)**	**0.008**
Self-rated health status^4^ (1–100)	68.8 (0.2)	67.0 (0.5)	**1.8 (0.5 to 3.1)**	**0.008**
Back pain intensity during past week^4^ (NRS, 0–10)	4.4 (0.1)	5.0 (0.2)	**–0.6 (–0.7 to –0.4)**	**<0.001**
Leg pain intensity during past week^4^ (NRS 0–10)	2.8 (0.3)	3.4 (0.3)	**–0.6 (–0.6 to –0.5)**	**<0.001**
ÖMPSQ-short total score^4^	40.4 (0.2)	43.3 (0.2)	–2.9 (0.02 to 0.12)	**<0.001**
	Intervention % (n)	Control % (n)	OR (95% CI)	
ÖMPSQ-short risk groups*^5^				**0.039**
Low risk	47.1 (105)	46.6 (125)		
Medium	24.7 (55)	17.9 (48)		
High	28.3 (63)	35.4 (95)		
LBP frequency (daily LBP yes/no)^6^	44.8 3 (98)	48.5 (130)	**0.88 (0.78 to 0.99)**	**0.038**
Pain medication use ≥ 3 days during past week^6^	41.7 (93)	47.4 (127)	**0.73 (0.65 to 0.82)**	**<0.001**

SE: standard error; FABQ: Fear Avoidance Beliefs Questionnaire, higher values indicate increased fear-avoidance beliefs; ÖMPSQ: Örebro Musculoskeletal Pain Screening Questionnaire, higher values indicate higher risk of prolonged pain-related disability; PSEQ: Pain Self Efficacy Questionnaire, higher values indicate increased pain self-efficacy; RMDQ-24: Roland Morris Disability Questionnaire 24 form, higher values indicate more disability.

1 Mean (standard deviation), *p*-value for between-group difference from independent-samples *t*-test.

2 Percentage (number), *p*-value for between-group difference from χ^2^ test or Fisher’s exact test.

3 Median (interquartile range), *p*-value for between-group difference from Mann–Whitney U test. Missing data comprised 24.9% (*n*=74) in the intervention group and 24.9% (*n*=89) in the control group. LBP (low back pain), SBT (STarT Back Tool), DEPS (Depression Scale), scores 0 to 30, higher scores indicate more depressive symptoms.

4 Difference between intervention and control groups was analysed with linear regression using generalized estimating equations with exchangeable working correlation matrix. Positive and negative mean differences indicate higher and lower values among intervention group, respectively.

5,6 Difference between intervention and control groups was analysed with ordinal^5^ or binary^6^ logistic regression using generalized estimating equations with exchangeable working correlation matrix. Missing data in the intervention group comprised 24.9% (*n*=74) and in the control group 24.9% (*n*=89). ^Contacts with other professionals before and after study consent allowed.

* Low-risk (0‒39 points), medium-risk (40‒49 points), and high-risk (50‒100 points).

** FABQ (Fear Avoidance Beliefs Questionnaire) work – items 6, 7, 9, 10, 11, 12, 15.

*** FABQ physical activity – items 2, 3, 4, 5. Statistically significant findings highlighted in bold.

### Implementation fidelity

The first-contacting HCP differed significantly between the intervention and control groups (*p* < 0.001). A supportive phrase in the EPR system was marked among 97% of all those first HCP contacts that had a nurse as the first contact person. The SBT risk group was documented in the EPR for 79% of LBP patients in the intervention compared with none in the control group. The SBT-based care plan was documented for 67% of LBP patients in the intervention group. An active treatment plan was documented for 83% of LBP patients in the intervention compared with 54% in the control group (Table II).

**Table II:** Implementation fidelity (adherence to the intervention and intervention-related frequencies and percentages) at the first visit to healthcare professional (HCP)

Implementation fidelity outcomes	Intervention (*n*=297)	Control (*n*=357)	*p*-value
First contacting HCP* % (*n*)^1^			**<0.001**
Physician	11.4 (34)	29.4 (105)	
First nurse then physician	26.9 (80)	11.2 (40)	
Physiotherapist	54.2 (161)	47.9 (171)	
Nurse alone	1.7 (5)	0.6 (2)	
Supportive phrase used by a nurse in electronic patient registry^1^	25.9 (77)	0 (0)	
SBT risk group documented in electronic patient registry^1^	78.5 (233)	0 (0)	
SBT-based LBP care plan documented^1^	67.3 (200)	0(0)	
Active treatment plan documented^1^	82.7 (230)	54.2(163)	**<0.001**
Psychosocial and lifestyle aspects documented in electronic patient registry			
Sleep documented^1^	28.4 (79)	27.3 (83)	0.764
Mood documented^1^	13.7 (38)	7.2 (22)	**0.011**
Social aspect documented^1^	31.7 (88)	25.7 (78)	0.110
Physical activity documented^1^	46.8 130)	47.0 (143)	0.947
Smoking documented^1^	4.7 (13)	8.6 (26)	0.062
Number of documented psychosocial and lifestyle aspects^2^ Mean (SE)	1.4 (0.3)	1.3 (0.1)	0.431

SBT: STarT Back Tool; LBP: low back pain; SE: standard error.

1 Percentage (frequency), *p*-value for between-group difference from χ^2^ test.

2 Values are estimated least square means with standard error.

3 Difference between intervention and control groups was analysed with linear regression using generalized estimating equations with exchangeable working correlation matrix.

Missing data in the intervention group comprised 5.8% (*n*=17) and in the control group 10.9% (*n*=39).

* Nurses were supported to participate in LBP patients’ care before physician appointments to ensure the use of SBT, and the patient education booklet to ensure SBT risk group-based care.

All data from electronic patient registry at first study contact. Statistically significant findings highlighted in bold.

### Primary outcome

We found no difference between the intervention and control groups in the primary outcome, change in PROMIS PF-20 from baseline to 12-month follow-up (MD 0.33, 95% CI –2.27 to 2.9, *p* = 0.473).

### Secondary outcomes

The mean number of sick leave days was 4.4 in the intervention compared with 7.2 in the control group at 3 months (RR 0.58, CI 0.45 to 0.75; *p* < 0.001) and 9.9 compared with 17.5 at 12 months (RR 0.44, CI 0.26 to 0.74; *p* = 0.002) (Table III).

**Table III:** Healthcare use and number of sick leave days in the intervention (*N*=287) and control (*N*=307) groups

Outcome	Intervention	Control	Intervention vs control	Intervention vs control^a^
	Mean (SD)	Mean (SD)	RR (95% CI); *p*-value	RR (95% CI); *p*-value
Physician appointments^1^				
3 months	0.8 (1.1)	1.1 (1.4)	**0.65 (0.63 to 0.67); < 0.001**	**0.70 (0.65 to 0.75); <0.001**
12 months	1.2 (1.5)	1.7 (2.2)	**0.62 (0.58 to 0.67); <0.001**	**0.64 (0.54 to 0.76); <0.001**
Secondary health care appointments (Physiatrist + orthopaedist)^1^				
3 months	0.1 (0.5)	0.3 (0.9)	**0.42 (0.30 to 0.60); <0.001**	**0.41 (0.28 to 0.59); <0.001**
12 months	0.3 (0.8)	0.6 (1.4)	**0.53 (0.30 to 0.95); 0.034**	**0.56 (0.37 to 0.85); 0.006**
Physiotherapist appointments^1^				
3 months	1.4 (1.4)	1.3 (1.5)	1.12 (0.92 to 1.36); 0.255	1.22 (0.94 to 1.59); 0.129
12 months	1.8 (1.9)	1.9 (2.5)	0.97 (0.80 to 1.18); 0.770	1.07 (0.85 to 1.34); 0.581
Nurse appointments^1^				
3 months	0.4 (0.6)	0.5 (1.1)	1.28 (0.82 to 2.00); 0.278	1.44 (0.92 to 2.24); 0.108
12 months	0.4 (0.7)	0.5 (1.1)	0.95 (0.76 to 1.17); 0.620	0.93 (0.68 to 1.27); 0.653
Sick-leave days^1^				
3 months	4.4 (13.3)	7.2 (18.7)	**0.64 (0.53 to 0.77); <0.001**	**0.58 (0.45 to 0.75) <0.001**
12 months	9.9 (40.9)	17.5 (55.5)	**0.59 (0.36 to 0.97); 0.037**	**0.44 (0.26 to 0.74) 0.002**
	% (*n*)	% (*n*)	OR (95% CI); *p*-value	OR (95% CI); *p*-value
Imaging*^2^ (yes/no)				
3 months	7.3 (21)	15.0 (46)	**0.45 (0.41 to 0.50); <0.001**	**0.39 (0.26 to 0.59); <0.001**
12 months	15.7 (45)	25.7 (78)	**0.55 (0.45 to 0.67); <0.001**	**0.53 (0.41 to 0.68); <0.001**
Radiographs^2^				
3 months	3.5 (10)	7.8 (24)	**0.43 (0.25 to 0.73); 0.002**	**0.33 (0.17 to 0.65); 0.001**
12 months	5.7 (17)	12.4 (38)	**0.44 (0.41 to 0.49); <0.001**	**0.34 (0.26 to 0.43); <0.001**
Magnetic resonance imaging (MRI)^2^				
3 months	4.5 (13)	8.7 (27)	**0.48 (0.40 to 0.59); <0.001**	**0.44 (0.29 to 0.69); <0.001**
12 months	11.5 (33)	18.7 (58)	**0.57 (0.38 to 0.86); 0.007**	**0.54 (0.32 to 0.93); 0.027**
Computed tomography (CT)^2^				
3 months	0 (0)	1.0 (3)	Unable to calculate*	Unable to calculate*
12 months	0 (0)	1.6 (5)	Unable to calculate*	Unable to calculate*
MRI+CT^2^				
3 months	4.5 (13)	9.1 (28)	**0.46 (0.36 to 0.60); <0.001**	**0.41 (0.25 to 0.66); <0.001**
12 months	11.5 (33)	19.5 (60)	**0.55 (0.36 to 0.83); 0.004**	**0.51 (0.32 to 0.82); 0.005**
Back surgery^2^				
12 months	1.4 (4)	2.9 (9)	0.46 (0.21 to 1.04); 0.061	0.52 (0.20 to 1.32); 0.166

a Adjusted for number of comorbidities, smoking, age, gender, radicular pain, multisite pain, chronic pain, SBT risk group.

1 Difference between intervention and control groups was analysed with Poisson regression using general estimating equations with exchangeable working correlation matrix.

2 Difference between intervention and control groups was analysed with logistic regression using generalised estimating equations with exchangeable working correlation matrix.

RR: risk ratio; CI: confidence interval; OR: odds ratio. *Calculation of OR was not possible due to zero frequency.

Full intention-to-treat method was used in all repeated measures to assess differences over time between intervention and control groups.

* Imaging includes all imaging modalities (radiograph, MRI, and CT). Statistically significant findings highlighted in bold.

The mean number of physician appointments in primary care was lower in the intervention group compared with the control group over 3 months (RR 0.70, CI 0.65 to 0.75; *p* < 0.001) and 12 months (RR 0.64, CI 0.54 to 0.76; *p* < 0.001) (Table III). The mean number of secondary healthcare appointments was also lower in the intervention group compared with the control group both at 3 months (RR 0.41 CI 0.28 to 0.59; *p* < 0.001) and at 12 months (RR 0.56, CI 0.37 to 0.85; *p* = 0.006). We found no significant difference in mean number of physiotherapy or nurse appointments at 3 months or 12 months. In the intervention group, 1.4% of patients underwent back surgery by 12 months compared with 2.9% in the control group (RR 0.52, CI 0.20 to 1.32; *p* = 0.166).

The proportion of LBP patients who underwent any imaging examination was lower in the intervention group compared with the control group both at 3 months (7.3% vs 15.0%; OR 0.39, Cl 0.26 to 0.59; *p* < 0.001) and at 12 months (15.7% vs 25.7%, OR 0.53, CI 0.41 to 0.68; *p* < 0.001).

Change in PROMIS PF-20 from baseline to 12 months did not differ significantly over time between the intervention and control groups (*p* = 0.473), the mean difference at 3-month follow-up being 2.37 (95% CI 0.15 to 4.59) and 0.33 (95% CI –2.27 to 2.9) at 12-month follow-up (Table IV). Change in LBP intensity from baseline to 12-month follow-up differed over time between the intervention and control groups (treatment*time *p* = 0.003). Pain reduction was achieved already at the 3-month follow-up (MD –1.3, 95% CI –2.1 to –0.5) in the intervention group, while in the control group the same level of reduction was shown at 12 months (MD 0.7, 95% CI –0.2 to 1.5). Similarly, leg pain intensity recovered earlier in the intervention group (*p* = 0.004). Change in LBP-related disability using ODI from baseline to 3- and 12-month follow-ups did not differ between the intervention and control groups (MD –0.24, 95% CI –4.55 to 4.07, treatment*time *p* = 0.299) although at 3 months difference in ODI favoured the intervention group (MD –4.57, 95% CI –8.76 to –0.39). We found no difference in change in EQ-5D from baseline to 3- (MD 0.059, 95% CI –0.020 to 0.137) and 12-month (MD –0.048, 95% CI –0.137 to 0.040) follow-ups (treatment*time *p* = 0.193). At the 3-month follow-up self-rated health was better in the intervention group (MD 6.9, 95% CI 2.9 to 11.0) while it was similar in both groups at 12-month follow-up (MD –4.1, 95% CI –9.8 to 1.6), over time between-group difference being significant (treatment*time *p* = 0.004). The percentage of LBP patients with daily LBP symptoms decreased in the intervention from 44% to 33% and in the control group from 49% to 42% (OR 1.08, 95% CI 0.43 to 2.72, *p* = 0.025).

**Table IV:** Patient-reported outcomes in the intervention (*N*=297) and control (*N*=357) groups

Outcome	*N*	Intervention vs control				Intervention vs control^a^			
		Mean^1^ (SE) Intervention	Mean^1^ (SE) Control	Mean difference (MD)^2^ (95% CI) *	*p*-value**	Mean^1^ (SE) Intervention	Mean^1^ (SE) Control	Mean difference (MD)^2^ (95% CI) *	*p*-value**
PROMIS PF T-score^2^					0.247				0.473
Baseline	470	45.0 (0.57)	43.9 (0.51)	1.09 (–0.18 to 2.36)		46.3 (0.81)	45.2 (0.59)	1.13 (–0.60 to 2.85)	
3 m	324	46.4 (0.61)	46.3 (0.61)	0.08 (–1.41 to 1.58)		49.6 (0.91)	47.3 (0.88)	**2.37 (0.15 to 4.59)**	
12 m	281	46.7 (0.66)	46.6 (0.62)	0.15 (–1.44 to 2.01)		49.6 (1.22)	49.2 (0.91)	0.33 (–2.27 to 2.9)	
Low back pain intensity during past week (NRS, 0–10)^2^					**<0.001**				**0.003**
Baseline	491	4.6 (0.2)	5.1 (0.2)	**–0.46 (–0.91 to –0.01)**		3.9 (0.3)	4.6 (0.2)	–0.68 (–1.3 to –0.1)	
3 m	353	3.7 (0.2)	4.4 (0.2)	**–0.70 (–1.21 to –0.19)**		2.4 (0.3)	3.7 (0.3)	**–1.3 (–2.1 to –0.5)**	
12 m	316	3.8 (0.2)	3.4 (0.2)	0.39 (–0.14 to 0.92)		2.7 (0.4)	2.0 (0.3)	0.7 (–0.2 to 1.5)	
Leg pain intensity during past week (NRS, 0–10)^2^					**0.037**				0.058††
Baseline	491	2.9 (0.2)	3.4 (0.2)	–0.55 (–1.08 to –0.01)		2.7 (0.4)	3.3 (0.3)	–0.6 (–1.4 to 0.2)	
3 m	353	1.9 (0.2)	2.9 (0.2)	–0.93 (–1.48 to –0.39)		1.2 (0.3)	2.2 (0.3)	**–1.0 (–1.8 to –0.2)**	
12 m	316	2.3 (0.2)	2.4 (0.2)	–0.12 (–0.67 to 0.44)		1.8 (0.4)	1.5 (0.3)	0.4 (–0.4 to 1.2)	
ODI%^2^					0.471				0.299
Baseline	491	23.2 (1.2)	24.7 (1.1)	–1.47 (–4.11 to 1.15)		20.3 (1.5)	21.4 (1.2)	–1.17 (–4.44 to 2.09)	
3 m	343	19.0 (1.3)	19.9 (1.2)	–0.88 (–3.64 to 1.88)		12.8 (1.7)	17.3 (1.6)	**–4.57 (–8.76 to –0.39)**	
12 m	306	18.9 (1.3)	18.6 (1.3)	0.21 (–2.80 to 3.23)		12.3 (1.9)	12.5 (1.5)	–0.24 (–4.55 to 4.07)	
EQ-5D-3L index†^2^					0.318				0.193
Baseline	491	0.679 (0.02)	0.629 (0.02)	0.050 (0.006 to 0.093)		0.706 (0.03)	0.682 (0.02)	0.023 (–0.036 to 0.082)	
3 m	352	0.707 (0.02)	0.690 (0.02)	0.017 (–0.030 to 0.064)		0.775 (0.04)	0.716 (0.03)	0.059 (–0.020 to 0.137)	
12 m	316	0.718 (0.02)	0.697 (0.02)	0.021 (–0.029 to 0.071)		0.752 (0.04)	0.800 (0.03)	–0.048 (–0.137 to 0.040)	
Self-rated health EQ-VAS (0–100)^2^					0.380				**0.004**
Baseline	486	67.3 (1.6)	65.9 (1.5)	1.4 (–2.3 to 5.1)		72.7 (2.0)	72.2 (1.6)	0.52 (–3.8 to 4.9)	
3 m	345	69.7 (1.7)	71.2 (1.5)	–1.5 (–5.3 to 2.3)		83.1 (1.8)	76.2 (1.8)	**6.9 (2.9 to 11.0)**	
12 m	312	71.6 (1.6)	71.9 (1.7)	–0.3 (–4.3 to 3.7)		79.0 (2.7)	83.1 (1.9)	–4.1 (–9.8 to 1.6)	
	*N*	Intervention % (*n*)	Control % (*n*)	OR (95% CI)				OR (95% CI)	
		Daily LBP yes	Daily LBP yes	1.02 (0.92 to 1.12)	0.295			**1.08 (0.43 to 2.72)**	**0.025**
LBP frequency (daily LBP yes/no)^3^ (binary logistic)									
Baseline	489	44.3 (98)	48.5 (130)						
3 m	353	32.6 (58)	41.7 (73)						
12 m	316	28.8 (42)	30.6 (52)						

a Adjusted stepwise for 1) number of comorbidities, smoking, age, gender, 2) radicular pain, multisite pain, chronic pain, and 3) StarT Back Tool (SBT) risk group Presented as intervention group vs control group.

1 Values are estimated least square means with standard error.

2 Difference between intervention and control groups was analysed with linear regression using generalized estimating equations with exchangeable working correlation matrix. Positive and negative mean differences indicate higher and lower values among intervention group, respectively.

3,4 Difference between intervention and control groups was analysed with binary^3^ or ordinal^4^ logistic regression using generalized estimating equations with exchangeable working correlation matrix.

Baseline has been measured *c*.1 to 3 weeks after the first visit to the healthcare professional. Baseline measure was not adjusted in the pairwise analyses at each time point, because the baseline values were affected by the intervention.

* Pairwise comparison has been made at each time point.

** *p*-values describe the statistical significance of the difference in change over time between intervention and control groups (time*intervention).

† EQ-5D UK TTO version;

†† Adjustment for radicular pain was not made here.

Full intention-to-treat method was used in all repeated measures to assess differences over time between intervention and control groups.

NRS: numerical rating scale; ODI: Oswestry Disability Index; PROMIS T-score: Patient-Reported Outcomes Measurement Information System, 20-item physical functioning short form T-score; EQ-5D-3L index: EuroQol 5 dimensions, 3-level version. Statistically significant findings highlighted in bold.

## DISCUSSION

### Key findings

Although classification-based care for low back pain patients in primary care did not appear to have an effect on physical functioning at 12 months, LBP-related healthcare use, imaging, and sick-leave days appeared to be lower both in the short (over the first 3 months) and long term (over the first year). In addition, LBP patients in the intervention group appeared to recover sooner compared with the control group.

### Comparison with previous literature

An earlier systematic review and meta-analyses suggested that a stratified care approach provided substantial clinical, economic, and health-related cost benefits in the medium- and high-risk subgroups compared with the usual care at 3, 4, and 6 months (30). Better adherence to recommended care was associated with less work disability in a retrospective cohort study (31). Our study shows that, using classification-based care, LBP patients recover sooner and the benefits of classification-based care in terms of reduced healthcare use and work disability remain over 12 months. Previously, successfully stratified care according to the SBT risk group has been shown to be effective (32), while non-successfully stratified care was not (33). In a recent US study among acute/subacute primary care back or neck pain patients, SBT-based stratified care provided a small reduction in pain-related disability at 3 months but no savings in healthcare use (34). Problems in that cluster-randomized study might be due to inconsistent information between “study HCP” and “usual care HCP” or an overly rigid protocol not allowing individualized care plans provided by skilled HCPs (35). Education of the professionals encompassed the whole organization, enhancing concordant communication between HCPs and patients in our study. Support of healthcare organization leaders was ensured to enable implementation, which was adapted to the existing clinical context with no need for extra resources. The use of supportive phrases by nurses was advanced by customizing the phrases according to the intervention. Reminders and structural changes within a healthcare organization have the potential to enhance implementation of a new protocol in practice (35, 36). HCPs had easily available printed versions of patient education booklets. An earlier cluster-randomized study suggested that systematic use of the patient education booklet reduced the mean number of sick-leave days and imaging rates by a similar magnitude to this study, while use of a patient education booklet did not appear to have an effect on number of physician appointments or pain relief (12). Many parts of the implementation really were successful and measurable (use of SBT, classification-based care plan, increased direct access to physiotherapy, nurse support for physician visits). It is possible that the most effective part of implementations might have been the booklet with evidence-based information on, e.g., the role of imaging, increased direct access to physiotherapy, routine referral to physiotherapy for medium- and high-risk patients thus bypassing the usually long waiting times for high-risk patients, and ensuring this would happen to as many LBP patients as possible through EPR phrases used by nurses. According to previous studies, education might not have been extensive enough to achieve fully adequate skills for all physiotherapists to deliver psychologically informed physiotherapy (37, 38).

### Interpretation of findings

Classification-based care for LBP in primary care does not appear to be effective in improving patients’ physical functioning, but it did appear to be effective in reducing LBP-related healthcare use and sick-leave days and appeared to provide quicker pain reduction compared with the usual care. Although we targeted the HCP resources to appointments with nurses and physiotherapists as early as possible, instead of with physicians, to ensure appropriate classification-based care, the rates of nurse and physiotherapist appointments at 3 or 12 months were not significantly higher in the intervention group. However, the number of LBP-related physician appointments in primary care and referrals to secondary care were lower in the intervention group at both 3 and 12 months.

### Implications of the findings

We recommend classification-based care for LBP patients in primary care in similar healthcare systems. More studies in different kinds of healthcare systems are needed as the Finnish primary care context differs, e.g., from the Scandinavian system (39).

### Strengths and limitations

Strengths of this study include delivering the intervention in a real-life environment and extensive documentation of the implementation process. Our benchmarking controlled trial design allows staggered timing for each healthcare area. In this way we could facilitate learning from experiences of the new classification-based care during the intervention period. Because the intervention trainings were of similar duration, we also hoped that this would enhance balance in recruitment in the intervention arm. Organizational and competence biases were reduced by including 3 different healthcare areas in the study and using them first as a control area and, after implementation, as an intervention area. Inclusion criteria included acute, subacute, and chronic LBP patients, which enhances feasibility and generalization to system-level use of classification-based care among primary care LBP patients. Due to the complexity of the intervention, which included organizational-, HCP-, and patient-level components, it is not easy to find which parts of the intervention are the most valuable. For example, bypassing the long waiting times for high-risk patients might be important and relevant only in healthcare systems where waiting times would be a limiting step for care. Reliability of the data was enhanced by using ERP for healthcare use and implementation measures. The intervention was slightly adjusted to better fit each organization’s resources and treatment pathways, which could support successful implementation of classification-based care elsewhere (40). It is suggested that interventions tailored to identified barriers are more likely to improve professional practice (41). Also, the availability of printed material may have enhanced implementation (42).

Due to strict privacy protection and organizational limitations, patient-reported outcomes were not permitted to be collected before the first HCP visit and patients’ signed consent. This restriction and delay in baseline patient-reported data might have changed the observed differences between intervention and control groups.

Baseline characteristics after the first visit to an HCP (Table I) seem likely to have been affected by intervention or healthcare area due to differences in care pathways, skills of individual professionals, and use of the patient education booklet. We have attempted to minimize selection bias by using suitable selection criteria and three-phase adjustment in the analyses. Adjustment for pain intensity was also tested in sensitivity analyses, but it did not markedly change the results. The first study contact included the patient education booklet, which has been shown in earlier studies to reduce LBP-related fear and to encourage LBP patients to be physically active (13, 24). HCPs in the intervention group were also trained to improve patients’ self-efficacy and drug-free pain relief, and to encourage physical activity. Implementation fidelity measurements also show that the first visit in the intervention really was different compared with the respective visit in the control group. Individualized treatment according to the SBT was delivered only in the intervention group. Active treatment, the core of the evidence-based care for LBP, was offered more often in the intervention group. Thus, statistically significant differences between the groups in terms of the baseline questionnaire 1 to 3 weeks after the first study contact may well be differences due to the classification-based intervention, but should be interpreted with caution. High dropout in terms of patient-reported data is also a limitation of the study. As can be seen from Fig. 2, a significant amount of missing data for recruited patients could add a major source of uncertainty to the reported results. Unfortunately, we do not have data with which we could attempt to try and correct missingness in a robust or comprehensive manner. This BCT study facilitates analysis with a broad range of real-world electronic-record data for recruited patients but reflects well-known challenges relating to response rates for questionnaires administered in routine healthcare settings. However, the response rates in this BCT are higher than in many other studies in Finland collecting similar data in routine healthcare settings (see, e.g., https://arviointikertomushus.fi/wp-content/uploads/2023/04/Arviointikertomus-2022.pdf, p. 40), and we suggest our study provides a useful starting point for assessment of routine effectiveness.

### Conclusions

Classification-based care for low back pain patients in primary care did not appear to influence physical functioning over 12 months. However, LBP-related healthcare use, proportion of LBP patients who underwent imaging examinations, and mean number of sick leave days appeared to be lower in the intervention group. Notably, LBP patients in the intervention group appeared to recover sooner compared with the control group. A classification-based approach for LBP patients in primary care seems to lead to favourable outcomes for the patients and the society.

## Supplementary Material

EFFECTIVENESS OF A CLASSIFICATION-BASED APPROACH TO LOW BACK PAIN IN PRIMARY CARE: A BENCHMARKING CONTROLLED TRIAL

EFFECTIVENESS OF A CLASSIFICATION-BASED APPROACH TO LOW BACK PAIN IN PRIMARY CARE: A BENCHMARKING CONTROLLED TRIAL

## Trial registration

ISRCTN, ISRCTN13273552. https://doi.org/10.1186/ISRCTN13273552.
