# Supplementary material for: EFFECTIVENESS OF A CLASSIFICATION-BASED APPROACH TO LOW BACK PAIN IN PRIMARY CARE: A BENCHMARKING CONTROLLED TRIAL
Source: J Rehabil Med. 2024 Apr 20;56:28321. doi: 10.2340/jrm.v56.28321 (PMC11151494; doi:10.2340/jrm.v56.28321)

Supplementary material has been published as submitted. It has not been copyedited, or typeset by Journal of Rehabilitation Medicine

Fig. S1. Patient level collection of reported data in the control and intervention arms. The flow chart shows the time point of the first low back pain-related visit to healthcare professional in the study and the time of signed consent. There was a delay of 1 to 3 weeks from the first contact with a healthcare professional in the patient's own primary care center and collection of baseline questionnaire data requested by the research nurse.

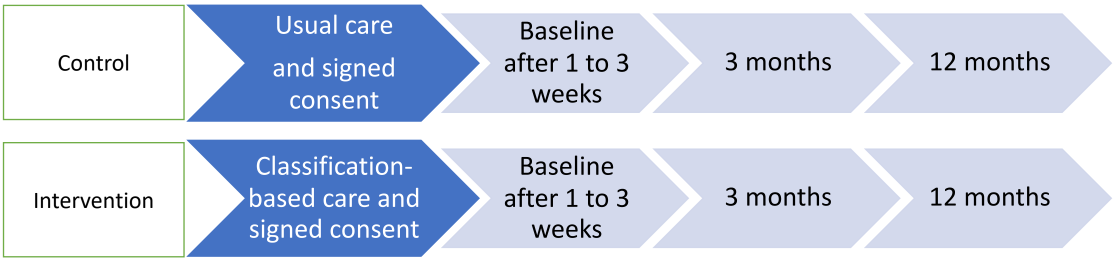

Supplement: EFFECTIVENESS OF A CLASSIFICATION-BASED APPROACH TO LOW BACK PAIN IN PRIMARY CARE: A BENCHMARKING CONTROLLED TRIAL [file JRM-56-28321-s1.pdf]
