# Supplementary material for: EFFECTIVENESS OF A CLASSIFICATION-BASED APPROACH TO LOW BACK PAIN IN PRIMARY CARE: A BENCHMARKING CONTROLLED TRIAL
Source: J Rehabil Med. 2024 Apr 20;56:28321. doi: 10.2340/jrm.v56.28321 (PMC11151494; doi:10.2340/jrm.v56.28321)
Supplement: EFFECTIVENESS OF A CLASSIFICATION-BASED APPROACH TO LOW BACK PAIN IN PRIMARY CARE: A BENCHMARKING CONTROLLED TRIAL [file JRM-56-28321-s2.pdf]

Supplementary material has been published as submitted. It has not been copyedited, or typeset by Journal of Rehabilitation Medicine

Table SI. A checklist for the electronic patient record data.

ID		
Date of consent		
First contacting professional: 1) physician; 2) first nurse and then physician; 3) physiotherapist; 4) nurse alone		
**Number of LBP related appointments**	**3 months**	**12 months**
Physiotherapist		
Nurse		
Physician		
Physiatrist		
Ortopedic surgeon		
All primary care contacts		
Number of LBP-related sick leave days		
Radiograph (X-ray)	yes/no	yes/no
Magnetic resonance imaging	yes/no	yes/no
Computed tomography	yes/no	yes/no
**Evaluation of implementation according to patient registry documentation from baseline contact (=date of consent)**		
Pain characteristics		
Presence of radicular pain	Yes / No /Unknown	
Presence of muscle weakness	Yes / No /Unknown	
Presence of cauda equina symptoms	Yes / No /Unknown	
Pain intensity (NRS)		
Physical functioning documented	Yes / No /Unknown	
STarT Back Tool (SBT) used	Yes / No	
SBT risk group	Low-risk / Medium-risk / High-risk	

Care plan according to risk group	Yes / No
Lifestyle and psychosocial factors documented	Yes / No
Mood	Documented /no documentation
Social situation	Documented /no documentation
Sleep	Documented /no documentation
Physical activity	Documented /no documentation
Smoking	Yes / No /Unknown
Goal setting for care plan	Documented /no documentation
Any physical activity care plan	Yes / No
Referral to physiotherapy	Yes / No
NRS: Numeral Rating Scale 0-10	

Table SII. STarT Back Tool (SBT) scores and risk groups.

Outcome	Time	Risk group	N	Interven-tion	Control	P-value
*SBT total score[1]	Baseline			4.5 (2.2)	4.9 (2.3)	
SBT risk group[2]						
	first study contact	low % (n);		22.9 (54);	not available	
		medium % (n);		47.0 (111);		
		high % (n)		30.1 (71)		
	Baseline	low % (n);	491	32.7 (73);	29.1 (78);	0.068
		medium % (n);		46.6 (104);	39.6 (106);	
		high% (n)		20.6 (46)	31.3 (84)	
	12 months	low % (n);	315	66.2 (96);	66.5 (113);	
		medium % (n);		20.7 (30);	18.8 (32);	
		high % (n)		13.1 (19)	14.7 (25)	

Presented as intervention group vs control group. [2]Difference between intervention and control groups was analyzed with ordinal logistic regression using generalised estimating equations with exchangeable working correlation matrix.

*P-value describes the statistical significance of the difference in change from baseline to 12 months between intervention and control groups (time*intervention).*

** The maximum total score range will be 0–9, subscale will be derived from Questions 5–9 (range 0–5): 1) Low-risk (total score of 3 or less); 2) Medium-risk (total score of 4 or more and psychosocial subscale score of 3 or less); 3) High-risk (total score and psychosocial subscale score of 4 or more).*
